# Event and Comment

**Published:** 1908-01-15

**Authors:** 


### DENTAL REGISTER.

**Vol. LXII. January 15, 1908. No. 1.**

### EVENT AND COMMENT.

### "Silk versus Calico."

At the recent meeting of the Ohio State Dental Society a paper was read under the above title. It was humorously stated and largely discussed in the same manner, and yet it called up a most interesting and serious problem which the profession has been compelled to consider throughout its history, and one which will probably not be settled satisfactorily by this discussion, or in the near future. The question raised, in brief, was whether an honorable or ethical practitioner was justified in dispensing an inferior service to one who could not pay what he considered a fair recompense for his service, and was justified in exacting a larger fee for a better service from such as could afford to pay. As the matter was put, it was "*calico for the poor and silk for the rich.*" On one side it was maintained that plastic fillings meet the needs of the poor and do not encroach unreasonably on the time and opportunity of the dentist; while gold fillings and inlays are within the means of the well-to-do class, and afford fitter recompense to the dentist, and exact a higher order of skill, therefore more to be desired and cultivated.

Every question of this sort has a moral precedent, and should be judged only upon that basis. It is the so-called practical factor that makes all the confusion and difficulty in adjusting such problems. The moral basis is unquestionably the right one, but unfortunately it will not pay rent, or buy automobiles. Dentistry is undoubtedly a beneficient profession, but as yet it can lay no large claims to being a philanthropic calling. We all feel kindly toward the unfortunate, and no dentist would hesitate to relieve any person, however destitute, from suffering, and at considerable cost; but it would be suicidal for him to give largely of his time and strength to such deserving charity, unless he had some other adequate means of support. As the greatest number of practitioners are dependent themselves upon their professional resources for their daily bread, it is evident that no great amount of charitable work can be indulged in, however benevolently inclined one may be. This problem is being worked out by the medical profession through the "out-door dispensaries" for the very poor, and public hospitals for such as may not be able to command the services of the regular practitioners. In some places the public dental clinic is being utilized to take care of these patients to some extent at least. But it will be a long time before the public authorities will provide generally adequate facilities for dental services of this character.

It is said that a French Prime minister once called an eminent surgeon to treat him, and remarked to the surgeon that he wanted his most skillful treatment, and not such as he was accustomed to deal out to the unfortunates whom he served in the public hospitals. The eminent surgeon replied that to him every patient he served was a Prime Minister. This undoubtedly should be our attitude towards all who seek our aid, and it may come to pass in the good time that is coming, and perhaps it should be our greatest ambition to strive to bring on such a beneficient condition by resolving that "all coons shall look alike to us."

We once visited a very skillful dentist who took us into his laboratory to show us a very fine piece of bridge work that he was preparing for the mouth of a wealthy railroad president. We admired the work very much, as it was very skillfully devised and constructed. The dentist informed us that he originally expected to charge $350 for the work, but as the patient was able to pay, and wanted the best service he could give him, he had, as he put more work and thought into the work, gradually worked his fee up to $700. We felt that in so doing under the circumstances he was perfectly justified in increasing the fee he would charge because he was actually putting the value into the service. But to our disgust, he informed us that his wife had just been in, and had plead with him for $50, which she wanted that she might purchase a fine velvet coat for their little girl, he finally gave it to her, and at the same time mentally agreed that he would add the fifty dollars to his fee for the bridge. We left that office with the feeling that our calling was unworthy of the name of a profession, and that if it was not actually dishonored by this action, it was so closely allied with the bad features of commercialism by a man of high abilities, whom the public was willing to trust, that we blushed for shame, and were filled with indignation that any man should so debase it.

We know another skillful practitioner who believes that a professional man has no moral right to take more of a rich man's money for a similar service than a poor man's. He carries his theory out in practice, and makes all his fees on the basis of so much per hour, and says his patients can make out their own bills as well as he can if they will take the trouble to keep track of the time they are in his chair, as they all know the rate per hour. Such a method, when practicable, has much to commend it, but there is a public responsibility attaching to a service rendered to one individual that does not apply to another, which makes the hour system unfair to the dentist as well as to the patients served. All patients do not make the same demands on one's physical or technical resources, and the responsibility in one case can never be the same as in another. Hence the value of the service can not be measured by a clock, but should be assessed on an intelligent, as well as a moral basis.

In the average practice it will always be necessary to make discriminations as to the kind of service to render in each case. In one case it may be of the calico order, and in another of the silk; but the quality in either case must be of the professional standard, and be based on an intelligent moral conception of professional duty and dignity; otherwise we shall never deserve the title of a profession, and may degrade our calling to the level of the most depraved commercial practices. There undoubtedly are some in our profession who have never realized the professional code, and others who condemn it outright, and still others who feel called upon to ignore it in order that they may succeed in a business way; but we believe the larger number of dentists realize that they hold an important position of trust, and they are loyally endeavoring to serve the people faithfully and conscientiously. Let us keep up our ideals and standards, and hope for the great day when all shall see that their true happiness and welfare is best secured by a constant loyalty to the highest attainments possible, and with their completest administration to all who require them.

